# The feasibility of a web-based counselling program for occupational physicians and employees on sick leave due to back or neck pain

**DOI:** 10.1186/1472-6947-9-46

**Published:** 2009-11-06

**Authors:** Tanja de Jong, Judith Heinrich, Birgitte M Blatter, Johannes R Anema, Allard J van der Beek

**Affiliations:** 1 Body@Work, Research Center on Physical Activity, Work and Health TNO-VUmc, Amsterdam, the Netherlands; 2 TNO Quality of Life, Hoofddorp, the Netherlands; 3 Department of Public and Occupational Health, EMGO Institute VUmc, Amsterdam, the Netherlands; 4 Research Center for Insurance Medicine AMC-UWV-VUmc, Amsterdam, the Netherlands

## Abstract

**Background:**

The objective of this feasibility study was to gain insight into occupational physicians' (OPs) and employees' use of, and attitudes towards, 'Snelbeter' (Get Well Fast), a new web-based counselling program for employees on sick leave due to non-specific back or neck pain and their OPs.

**Methods:**

Registered user information was collected from the website to get insight in the use of the program by employees (n = 24). Qualitative information was obtained through semi-structured in-depth interviews with 19 OPs and nine employees in order to get insight in the actual use of the provided information, the attitudes towards the program and possible improvements of the program.

**Results:**

Actual use of the program among OPs was low. The majority of OPs, eight out of 11 (73%), never or only occasionally signed in. The greatest obstacle for OPs to use the program was the low number of eligible employees involved. Employees appreciated the program but their use was moderate. A small majority of the employees who used the program, 14 out of 24 (58%), opened 50% to 100% of the provided documents, a majority of the interviewed employees, seven out of nine (78%), used the provided information sometimes or regularly. The absence of personal contact was found to be a major barrier towards use of the program by employees.

**Conclusion:**

Although both OPs and employees appreciated the idea of the program and employees appreciated using it, program utilization was moderate to low. The discussion section reveals that before implementation can be started to any extent, the program will need adaptations that make it more attractive to use. The program should be considered for both return to work (RTW) and the prevention of sick leave. Adding personal contact (e.g. involving physiotherapists) to the program may also be promising.

## Background

The most prevalent musculoskeletal symptom in the Netherlands is low back pain (12-month prevalence 44%), followed by neck pain (12-month prevalence 31%) [1]. Musculoskeletal symptoms show an episodic pattern and in most cases improve spontaneously over time, without medical intervention or sick leave [1]. If sick leave due to back pain does occur, it is often for short periods only since 82% of all employees return to work within a month [2]. Neck pain also has a good prognosis; almost half of the employees on sick leave due to neck pain return to work within a week [3]. Although both back and neck pain have a good prognosis for the majority of employees, any delay in return to work (RTW) results in high compensation and treatment costs [4,5]. Obviously, the prevention of long-term sick leave is important. The literature mentions various issues regarding interventions for the prevention of long-term sick leave due to back or neck pain. First, from the employee's perspective, improving self-efficacy with regard to RTW seems to be a promising factor [6,7]. Recovery expectation, a construct closely related to self-efficacy, has also been shown to be an important prognostic factor for sick leave [8]. Secondly, improvements could be made in the professional guidance of occupational physicians (OPs). Research and practical experience reveal that many OPs do not have a pro-active attitude to referring patients with non-specific back or neck pain to second-line care, even if they are maximally facilitated [9]. Thirdly, most likely the internet will obtain a more important role for occupational health care in the future, since it may save time for health professionals, save money for employers, and offers flexibility for employees. Although internet-delivered cognitive-behavioural interventions are a promising complement to existing treatments [10], this medium has seldom been used in occupational health care for employees suffering from back or neck pain.

To incorporate these factors in the usual care of employees on sick leave due to non-specific back or neck pain, a web-based counselling program named 'Snelbeter' (Get Well Fast) was developed. The purpose of this web-based counselling program (called 'the program' hereafter) is twofold: 1) to stimulate the self-efficacy of employees, and 2) improve the knowledge and capacity of OPs to deliver individualized care and support their referral process. The program was developed by two experts, an OP and a physiotherapist, after several discussions in the multidisciplinary research team. Before OPs could start, they had to follow a training in the purpose and use of the program.

The objective of this feasibility study is to gain insight into OPs' and employees' use of and attitudes towards the program. This paper answers the following questions:

1. To what extent did OPs and employees use the program?

2. What did OPs and employees appreciate about the program?

3. Did anything prevent the OPs and employees' from using the program?

4. How do OPs and employees think the program could be improved?

## Methods

### Description of the web-based counselling program

The program is accessible for the employee and his OP by http://www.snelbeter.nl[11]. The employee can log-in to consult information in a personal diary. Throughout the five weeks of the program, employees have to fill in four questionnaires. The questionnaires contain questions about pain, limitations, treatment, counselling, reintegration, the work situation and work characteristics, relations at work, personality and daily activities. Provision of information to the employee is based on the questionnaires. The information includes advice on how to improve physical fitness, how to set a daily timetable, how to use pain-coping strategies, and instructions for neck and back exercises. The OP also has access to the employee's personal diary. He receives an advisory report each time an employee completes a questionnaire. The report provides information about sickness absence, experienced complaints by the employee, current treatment and test results concerning pain, relations at work and competences. The reports advice the OP about medication, referral to second-line care and RTW. The characteristics of the program are summarized in table 1.

**Table 1:** Description of the web-based counselling program 'Snelbeter' (Get Well Fast)

*Target group*	Employees on sick leave due to back or neck pain, their occupational physicians (OPs) and (optionally) their direct supervisor.
*Inclusion criteria*	Non-specific back or neck pain, contract duration of 12 hours minimum per week, two weeks minimum (partly) on sick leave due to back or neck pain, no serious health problems (warning flags: e.g. fever, pain in arms or legs, serious disease), able to talk and write in Dutch and with access to the internet. In the case of full absenteeism, employees need to have internet access at home and a private e-mail address.

*Concept*	The program is based on self-reported questionnaires. Employees receive individually tailored instructions for exercises, pain education and coping tools. The information provided comes from the latest scientific knowledge concerning treatment and interventions for employees on sick leave due to non-specific back or neck pain. It also conforms to the Dutch practice guidelines for employees with neck and back complaints of the Netherlands Society of Occupational Medicine (NVAB) [17,18].
	
	The program offers OPs information with regard to second-line care referrals and return to work (RTW). The employee has the option to involve his supervisor. If the employee gives permission, the supervisor will receive information on how to counsel the employee.

*Time path*	The program takes five weeks from start to end and can be continued even if RTW is achieved. The time path of the program is in line with the NVAB's practice guidelines and includes these steps:
	1. Schedule daily physical activity;
	2. Define and list problems;
	3. Establish and list solutions;
	4. Go back to work.

*Information for employees*	Employees log in to the website to consult the information in a personal diary. Throughout the five weeks of the program, employees regularly receive e-mails reminding them to keep on visiting the website, even if they have fully returned to work. Employees may receive up to 14 documents in total, depending on the data derived from four questionnaires. The information includes advice on how to improve physical fitness, how to set a daily timetable, pain-coping strategies, and instructions for neck and back exercises. It takes employees about 15 minutes a day to read the documents, fill in the questionnaires and follow the exercises. If an employee refrains from filling in a questionnaire no more information is provided to that employee and the OP concerned.

*Information for OPs*	The OP receives an advisory report each time an employee completes a questionnaire. The OP receives an alert three days after a questionnaire is presented to the employee in order to contact those who have not yet responded. The OP also has access to the employee's personal diary.

### Design and study population

The feasibility study was conducted by gathering registered user information of the web-based program and interviewing both OPs and employees. The study population consisted of OPs working for either KLM or National Railways and employees of both companies absent due to back or neck pain. In the Dutch system, all employees are attached to an occupational health service which requires those on sick leave for longer than a fortnight to see their OP. During the first consultation, employees who met the inclusion criteria (see table 1) were invited to use the program. All employees (n = 24) who used the program were invited to an interview. The OPs who recruited employees (n = 11) as well as those who did not (n = 15) were also invited to an interview. It was emphasized that participation was fully voluntary. All answers would be treated confidentially and could not be traced back to individuals. OPs and employees agreed to participate in the study. The Medical Ethics Committee of the VU Medical Centre, the Netherlands, approved the study design, protocols, procedures and informed consent procedure.

### Data collection

To obtain quantitative insight into program use we collected registered user information on all 24 employees about the number of completed questionnaires and opened documents, irrespective of whether the information was actually read or used in practice. Information on the actual use of the program was extracted from the interviews. Between April and July 2008, OPs and employees were interviewed by telephone for about 30 minutes using an in-depth semi-structured format. The interviewers used three formats; a format for employees, a format for the OPs who recruited employees, and a format for those who did not recruit any employees. Table 2 shows a summary of the interview topics and questions. A pilot interview in each group tested the usability and feasibility of the format, and this led to slight adaptations. Interviews were recorded with the permission of the participants. The interviewer entered the answers to each topic in an Excel file and completed input after listening to the recording. The description of the answers included quotes providing qualitative insight into the opinions of the respondents. Both interviewers performed random checks on three of each other's interviews in order to verify whether the answers were interpreted consistently.

**Table 2:** Topic list for interviews

Occupational physicians (OPs)	Employees
**Use of the program**	**Use of the program**
*(only for OPs who recruited employees)* How often did you log-in to the program? Did you follow the employee's personal diary? Did you read the advisory reports sent to you? Did you receive recommendations for employees?	How often did you read the program documents? Did you follow the offered advice? Did you do the exercises?
	
**Appreciation**	**Appreciation**
*General attitude towards the program*	*General attitude towards the program*
Did the program add value to your counselling of employees with back or neck pain (only for OPs who recruited employees)? Do you think a website is a good medium for counselling employees with back or neck pain? Do you think employees find the program useful?	Did the program add value to the treatment of your back or neck pain? Do you think a website is a good medium for treating your back or neck pain?
	
*Content*	*Content*
Is the program content clear? Is the content useful? Were any subjects missing from the program?	Is the program content clear? Is the content useful? Were any subjects missing from the program?
	
*Presentation*	*Presentation*
Is the program well-designed and user friendly?	Is the program well-designed and user friendly?
	
*Employee recruitment*	*Perceived effectiveness*
How many employees did you see per month that satisfied the inclusion criteria? Did you always have the program in mind when you met employees who satisfied the inclusion criteria? How did employees react to your recruitment invitation? Did you experience problems in recruiting employees? What problems?	Did the program help you communicate better with your OP about your pain? Did the program extend your knowledge about ways of coping with pain? Did the program help you to get well fast and return to work sooner than you may have otherwise?
	
**Perceived usage barriers**	**Perceived usage barriers**
Did you have any problems using the program?	Did you have any problems using the program?
	
**Improvements**	**Improvements**
Do you have any suggestions for improving the content? Do you have any suggestions for improving the design? Do you have any suggestions for improving the website in order to improve the use of the program?	Do you have any suggestions for improving the content? Do you have any suggestions for improving the design? Do you have any suggestions for improving the website in order to improve the use of the program?

### Data analysis and description of results

Quantitative data about the use of the program were analysed using count variables and percentages. Qualitative analysis involved the systematic examination and organization of data to identify themes related to each interview topic (appreciation, perceived usage barriers and improvements). Themes included the opinions and issues mentioned by one or more respondents as summarized by the researcher. Themes were identified through a crystallization process. Various key words were linked to the answers from OPs and employees. Then these were categorized into the themes. Simple counts were used to provide a summary of the results. The results are illustrated by quotations from participants.

## Results

### Study population

All 11 OPs who recruited employees participated in the interviews. Of the 15 OPs who did not recruit employees, eight participated in the interviews. Lack of time and personal considerations were reasons given for non-participation. We obtained registered user information for all 24 employees who used the program but only nine were available for an interview. Fifteen employees were unavailable for an interview even after several attempts had been made to reach them. Reasons for non-participation ranged from 'no time' to 'insufficient use of the program' and 'problems with recalling experiences'. The interviewed employee population consisted predominantly of men (67%) between 40 and 50 years suffering from low back pain (75%). Both white and blue collar workers with various levels of education were recruited. Sick leave duration due to back or neck pain including partial absenteeism ranged from seven weeks to six months.

### Use of the program by OPs who recruited employees

Use of the program by OPs was low. Three of the 11 OPs never logged in to the website to follow the information or instructions. Five OPs signed in occasionally and only three did so regularly. Six of the 11 OPs tracked the employee's personal diary and read the employee reports based on the questionnaire data at least one time, which included specific recommendations for RTW and referral to secondary care. Only one OP used these recommendations in practice.

### Use of the program by employees

Both the registered and actual use of the program was moderate among the employees. Registered data revealed that seven employees, out of 24 employees who used the program, filled in all four questionnaires. Another 12 filled in two or three questionnaires and the remaining five just one questionnaire. Ten employees opened less than 50% of the documents provided. Six employees opened between 50-75% of the documents, four employees opened almost all of them and four employees did indeed open them all. The majority (n = 7) of the nine interviewed employees reported that they read the provided documents sometimes or regularly and performed one or more of the exercises.

### Appreciation of the program by OPs

Most OPs believed that the program is useful for employees. Some stressed that it would be most useful for employees who did not know how to cope with pain or were experiencing pain for the first time. Only half of the OPs indicated that the program had added value for themselves. Some OPs mentioned that the program offered additional support for them especially when an employee's recovery was making no progress. About half of the OPs indicated that a website is a good medium for the counselling of employees with back or neck pain. More than half of the OPs were positive about the content (e.g. information, exercises, instructions). It was suggested that the program could be useful as a reference source as it provides a good overview of information for OPs. It also supports the taking of systematic action. Finally, almost all OPs were positive about the user-friendliness and design of the program.

### Appreciation of the program by employees

The majority of employees replied affirmatively to 'Did the program add value to the treatment of your back or neck pain?' Overall, employees experienced the program as a supportive tool. The program stimulated them to perform exercises at home, helped them give structure to their daily life and gave them the feeling that they could take an active approach to their pain and RTW process. Furthermore, employees felt their situation was gaining attention. They were positive about the content, user-friendliness and web-based design of the program. They said the information was easy to understand, to the point, and conveniently arranged. About half of the interviewed employees said that the program extended their knowledge of coping with pain and enhanced communication with their OP. A few employees reported that the program helped them to return to work faster.

Of all the available information, the neck and back exercises were appreciated the most. Some employees involved their physiotherapist in the RTW program. One discussed the exercises with his therapist. In contrast, other employees noted that the program was most useful when they received no additional treatment. The program was perceived as useful if employees lacked knowledge, were experiencing pain for the first time or had less severe pain. This outcome was supported by comments from the OPs.

### Program usage barriers for OPs

Although about half of the OPs were positive about the added value, content and user-friendliness of the program, all encountered obstacles which limited their use of the program in actual practice. The greatest barrier for OPs was the low number of employees who met the inclusion criteria, especially for those who did not recruit employees. Several OPs mentioned that employees' fast recovery also had an impact on the low numbers involved. Furthermore, many employees were contacted later than the official term, about two weeks after the employee has become absent from work. By this stage employees may already be under treatment by their general practitioner or another therapist. Because of the low number of employees involved, OPs did not get round to using the program with any routine. As one OP said, 'It takes time to get used to the recruitment process and to using the program.' More than half of all OPs did not keep the program in mind during consultations and some had difficulty providing employees with accurate information about the program.

A second important barrier for OPs was the limited time available for introducing employees to the program and working with it as well. As another OP pointed out, 'We lack the time to do this kind of projects.' Several OPs mentioned that since they were already burdened by many administrative tasks they found the program more of an extra duty than something that offered added value. As well as these major barriers, OPs indicated other aspects which discouraged their use of the program. One OP explained that he was quite capable of managing the RTW process himself and did not need a program for additional support. Many preferred the more familiar therapies (e.g. physiotherapy) for their employees despite the fact that the program can work as a supportive tool. They preferred having personal contact with employees. One OP stated that he did not use the program because he did not believe in 'computer-based treatment' of physical pain. He explained, 'The ability to touch people is an essential element in the treatment of people with back or neck pain.' Some OPs had no affinity with the use of a web-based program in general and therefore preferred not to use this method. Finally, some OPs faced practical obstacles, such as log-in problems or no access to a computer or the internet in their consulting rooms.

### Program usage barriers for employees

The greatest barrier for employees against using the program was the availability of other treatment such as physiotherapy, which involves personal contact. Employees perceived the program as less relevant because they were already getting exercises from their physiotherapist. For some employees the exercises suggested by the program conflicted with the exercises given by the physiotherapist. They felt that some exercises would be performed better after consultation with a physiotherapist. In addition, because some of the advice and exercises were not specific enough, they did not apply to the employee's situation. It was remarkable that almost no employees talked about the outcome of the program with their OPs. The program was not a subject of discussion during consultations; no OPs actually referred to the program. One employee said, 'I expected more commitment from my OP". This did not encourage employees to use the program. Only a few employees considered the large amount of information as a barrier. One employee mentioned that the back or neck pain they were suffering from may have prevented them from sitting at a computer. A small number of employees either had 'problems with logging in' into the program or had 'no affinity with computers'.

### Improvements for OPs

Although OPs were generally positive about the user-friendliness and design of the program, some felt that further improving user-friendliness (functionality) might enhance its use. For example, it was suggested that it would be useful if the program was able to sort a list of employees in the same way as the OP's company system does. It should also be easier to register employees in the program. A helpdesk or some additional support would be helpful in case the OP had practical questions. Alternative tools, instead of the program, were also suggested. According to one OP, a website with a practical overview of information would be sufficient to satisfy the needs of OPs and employees. Some questioned whether the OP should be involved in the program at all. For example the direct supervisor or the physiotherapist might have a more prominent role in the promotion and use of the program. One OP mentioned the importance of focussing on the prevention of sick leave due to back or neck pain, in addition to focussing on the RTW process. This broader focus should not only be on sick leave prevention but also on how to work with pain. Additional content should be provided for this purpose. OPs also meet employees at an earlier stage, for example during an open consultation or during a general medical assessment. According to one OP, this would be a good starting point for recruiting employees to the program. OPs put forward that the direct supervisors should be more involved in the prevention of sick leave due to back or neck pain since their support is important.

### Improvements for employees

A couple of employees mentioned the importance of customizing the advice to an employee's personal needs. Circumstances can change over time; therefore it is important that the advice fits the employee's current situation. Employees mentioned that a feedback function on the program would be helpful, which was also mentioned by OPs. For example, it would give the employee the opportunity to contact a professional by telephone or e-mail. According to one employee, a personal meeting a couple of weeks after the program is finished would be useful for evaluating recovery. It was suggested that combining hands-on treatment by a physiotherapist with the web-based counselling program would provide optimal support. Increasing the involvement of OPs or physiotherapists may stimulate employees to use the program. Employees also suggested other improvements related to the program content and user-friendliness (e.g. shortening the questionnaires and adding illustrations and cartoons). A link on the company website would also enhance use of the program. One employee felt that more attention should be paid to the psychological distress experienced by employees suffering from back or neck pain. He explained, 'I missed information on how to deal with it mentally.' Another employee felt that more attention should be paid to the prevention of sick leave due to back or neck pain. For example, preventive exercises could be made available on the company's website.

The perceived barriers and possible improvements of the program are summarized in table 3.

**Table 3:** Overview of program usage barriers and possible improvements of the program

Occupational physicians (OPs)	Employees
**Occupational physicians (OPs)**	**Employees**
- Low number of employees who met inclusion criteria - Limited time during consult - Preference for more familiar therapies and personal contact with employee - Knowledge of OP is sufficient, no extra support needed - No affinity with internet/webbased tools - Practical obstacles (e.g. log-in problems, no acces to computer at work)	- Availability of other treatment which involves personal contact - Conflict between exercises provided by secondary care and the website - Advices and exercises do not fit personal situation - Large amount of information - Computer use not prefered during neck/back pain - Technical problems
	
**Improvements**	**Improvements**
*Content*	*Content*
- Make it more easy to register an employee -Use the same sorting system of employees as in the company - Adding contact function on the website - Change content into practical overview of information	- Shorter questionnaires - More illustrations - Link to website on company website - Contact function via the website - Customize the information more to employee's needs
	
*Additional improvements*	*Additional improvements*
- More prominent role for supervisor/physiotherapist - Focus on the prevention of sick leave and how to 'work' with pain.	- Involvement of the OP or other therapist - Additional personal meeting focused on the counselling process - Focus on prevention of sick leave - More attention to psychological distress
	
*Recruitment*	
- Recruitment of employees in earlier stage	

## Discussion

The results of this feasibility study showed that although OPs and employees are generally positive about the web-based counselling program in terms of content and user-friendliness, in practice usage was low, particularly among OPs. Some employees returned to work during the program which may have been why they stopped using it. Some barriers limited the use of the program. For example, both OPs and employees mentioned that they prefer therapies with personal contact. This finding is in line with results reported by Steele et al. [12]. Their process evaluation of an internet-based program to change physical behaviour reveals that participants in the internet group preferred face-to-face sessions. They would have performed better had they been able to report to someone who gave them personal reinforcement for their behaviour change. This raises the question of whether greater involvement by a therapist might have improved the usage by employees. In our study the involvement of the OP in the program was low. Employees experienced no support from their OP in using the program. In a meta-analysis of internet-based interventions for anxiety and depression, Spek et al. found that the effect of interventions with therapist support was considerably larger than the effect of interventions without a therapist [13]. A review by Copeland and Martin on different types of internet-programmed interventions with little or no direct therapist involvement showed that many of such studies have high dropout rates [14]. Postel et al. also stressed the importance of working with professional therapists in online treatment programs since the skill and good sense of the therapist are important factors in the efficacy of a given treatment [15]. Although these findings are based on research on E-therapy for mental health problems, we find the involvement of a therapist is also of importance for physical health problems as well. A physiotherapist could take on the role if the employee is undergoing therapy. Since the physiotherapist is actively involved in treating all stages of back and neck pain and is involved in the recovery process he may provide proper feedback to employees [16]. The ways in which the physiotherapist could be involved should be investigated.

An issue mentioned by OPs and one employee is to focus on the prevention of sick leave as well. Broadening the program's focus to the prevention of sick leave and working with back or neck pain would increase the target population and could improve usage by OPs and employees.

To our knowledge, our study is the first to use a customized web-based program in occupational health care for non-specific back or neck pain. A strength of the study was that the program was applied in a real-life occupational healthcare setting. Therefore, the generalizability of the results is good. A second strength is that the web-based content conforms to the Dutch practice guidelines for employees with back and neck complaints [17,18]. Finally, we would like to emphasize that our study is an example of how qualitative information can complement quantitative information on the use of web-based programs.

This study also has limitations. First, the value of the results derived from interviews with OPs who only received training without including any employees is questionable since their answers were based on their opinions, not on their use of the program. Furthermore, since we interviewed only a limited number of employees and OPs, we may have missed relevant information. We possibly did not achieve full saturation, especially among employees. A recollection bias also needs to be taken into account, since the interviews took place more than six months after the intervention. We controlled for this partly by giving the respondents the opportunity to look at the website before the interview. There may have been some response bias during the interviews as well. Participants may have provided more desirable feedback about the program when faced by the interviewer. However, prior to starting the interview, we stressed that it was of great importance to be honest. A selection bias may have occurred if the only participants were those people who were positive about the program. However, we included OPs and employees who used the program to different extents (i.e. based on the number of filled in questionnaires and opened documents).

Overall, our study stresses the importance of assessing needs of both employees and OPs in preparation for intervention development. Needs assessment and context analysis is a systematic study of the discrepancy between what is and what should be needed in a group and situation of interest. A program can only be effective if there is a real problem or need [19]. A rigorous evaluation of the barriers and facilitators for implementation on the level of the innovation itself, care provider and context, could tailor the program better to the needs of the users and target group [20,21]. The involvement of the target group in the program development is important. Earlier research reveals that systematic development of interventions and tailoring their content and format to the specifics of the target group and setting seems necessary to improve the effectiveness of patient care [22]. Our study confirms this finding, since the format of the intervention did not entirely satisfy the needs of OPs and employees.

More general research on the use of web-based programs for musculoskeletal disorders in occupational health care is desirable. Since our intervention study was limited to OPs and employees from two transport companies it would be interesting to apply the web-based program to other target groups, for example, in the health care sector.

## Conclusion

The results of this study showed that the use of the program was low among OPs. The majority of OPs, eight out of 11 (73%), never or only occasionally signed in. Only one OP used the provided recommendations for OPs about RTW and referral to secondary care of employees in practice. Although the majority of OPs appreciated the value of the program for employees, only half of the OPs experienced added value personally. All OPs perceived barriers against using the program in practice. The low number of employees involved, lack of time and a preference for usual care were reasons why the program was hardly ever used in consultations. According to OPs some adaptations might improve its user-friendliness (e.g. simplifying registration of employees and adding a helpdesk function). However, because of their limited use of the program we questioned whether the OPs should be extensively involved in the program. Therapeutic involvement of a therapist does seems important, but perhaps someone else could fill the role played by the OP, for example a physiotherapist.

The employees used the program to a moderate extent. A small majority of the employees who used the program, 14 out of 24 (58%), opened 50% to 100% of the provided documents, a majority of the interviewed employees, seven out of nine (78%), used the provided information sometimes to regularly. Most employees confirmed that the program offered added value in their consultations for back or neck pain. They also appreciated the content and design of the program. However, as for OPs, the availability of usual care was a barrier against using the program. Employees indicated that the involvement of a therapist, more opportunities for feedback, the ability to customize the program, more emphasis on emotional factors, and a link to the company website were all factors that would improve the program.

OPs and an employee mentioned the importance of focussing on the prevention of sick leave due to back or neck pain We concluded that broadening the program's focus to the prevention of sick leave and working with back or neck pain would increase the target population and could improve usage by OPs and employees.

## Competing interests

The authors declare that they have no competing interests.

## Authors' contributions

TJ conceived the study, participated in the design and coordination of the study, performed the data analysis and drafted the manuscript. JH conceived the study, participated in its design and coordination and drafted the manuscript. BMB, JRA and AJB conceived the study and helped to draft the manuscript. All authors read and approved the final manuscript.

## Pre-publication history

The pre-publication history for this paper can be accessed here:

http://www.biomedcentral.com/1472-6947/9/46/prepub
